# Supplementary material for: Complexome profiling of the Chlamydomonas psb28 mutant reveals TEF5 as an early PSII assembly factor
Source: Plant Cell. 2025 Mar 17;37(6):koaf055. doi: 10.1093/plcell/koaf055 (PMC12164590; doi:10.1093/plcell/koaf055)
Supplement: koaf055_Supplementary_Data [file koaf055_supplementary_data.zip › psb28_interactive.zip!/plots/intensity/qualityPlots/peptides/PSAD_Cre05.g238332.t1.1.html]

### Cre05.g238332.t1.1

Trivial name: PSAD  
SeqCoverage [median]: 65.80  
Mass: 21.34 kDa  
  
 

| Sequence | MissedCleavage | Mass [Da] | wtIntensity | muIntensity | Score |
| --- | --- | --- | --- | --- | --- |
| AEAEAAPAAAK | 0 | 998.50 | 1292.97 | 0.00 | 90.66 |
| AQTEEFYVITWEAK | 0 | 1713.83 | 40499.05 | 0.00 | 221.60 |
| AQTEEFYVITWEAKK | 1 | 1841.92 | 166024.80 | 31269.98 | 152.17 |
| EQIFEMPTGGAAIMR | 0 | 1649.79 | 537112.31 | 600121.91 | 255.24 |
| IGQNVNPIK | 0 | 981.56 | 1306943.00 | 1465922.94 | 257.11 |
| IGQNVNPIKVK | 1 | 1208.72 | 223151.44 | 602421.77 | 219.56 |
| IGQNVNPIKVKFSGR | 2 | 1655.95 | 1343.08 | 1854.90 | 45.86 |
| KAQTEEFYVITWEAK | 1 | 1841.92 | 265573.54 | 214177.32 | 216.00 |
| KAQTEEFYVITWEAKK | 2 | 1970.01 | 147266.69 | 110813.48 | 228.49 |
| KEQIFEMPTGGAAIMR | 1 | 1777.89 | 1022555.94 | 669964.85 | 209.93 |
| MMSPAEI | 0 | 777.34 | 273247.57 | 346741.69 | 130.65 |
| QGPNLLK | 0 | 768.45 | 1067223.22 | 716610.41 | 134.12 |
| RIGQNVNPIK | 1 | 1137.66 | 242471.03 | 220221.07 | 166.07 |
| RIGQNVNPIKVK | 2 | 1364.83 | 2454.30 | 8315.99 | 107.06 |
| VGANQNMR | 0 | 888.42 | 865002.74 | 903895.16 | 206.10 |
| VGANQNMRR | 1 | 1044.52 | 6662.43 | 0.00 | 77.92 |
| VQYLHPADGVYPEK | 0 | 1614.80 | 3458.70 | 0.00 | 126.12 |
| VQYLHPADGVYPEKVNAGR | 1 | 2112.08 | 2262.07 | 0.00 | 53.28 |
|  |  |  | 6174544.9 | 5892331.5 |  |
